# Engineering caveolin-mediated endocytosis in *Saccharomyces cerevisiae*

**DOI:** 10.1016/j.synbio.2022.06.008

**Published:** 2022-07-02

**Authors:** Qian Zhang, Ning Li, Yunbin Lyv, Shiqin Yu, Jingwen Zhou

**Affiliations:** a Science Center for Future Foods, Jiangnan University, 1800 Lihu Road, Wuxi, Jiangsu, 214122, China; b School of Biotechnology, Jiangnan University, 1800 Lihu Road, Wuxi, Jiangsu, 214122, China; c Engineering Research Center of Ministry of Education on Food Synthetic Biotechnology, Jiangnan University, 1800 Lihu Road, Wuxi, Jiangsu, 214122, China; d Jiangsu Province Engineering Research Center of Food Synthetic Biotechnology, Jiangnan University, 1800 Lihu Road, Wuxi, Jiangsu, 214122, China

**Keywords:** Oil, Caveolin-1, β-Oxidation, (2*S*)-Naringenin, *Saccharomyces cerevisiae*

## Abstract

As a potential substitute for fatty acids, common low-cost oils could be used to produce acetyl-CoA derivatives, which meet the needs of low-cost industrial production. However, oils are hydrophobic macromolecules and cannot be directly transported into cells. In this study, caveolin was expressed in *Saccharomyces cerevisiae* to absorb exogenous oils. The expression of caveolin fused with green fluorescent protein showed that caveolin mediated the formation of microvesicles in *S. cerevisiae* and the addition of 5,6-carboxyfluorescein showed that caveolae had the ability to transport exogenous substances into cells. The intracellular and extracellular triacylglycerol levels were then detected after the addition of soybean oil pre-stained with Nile Red, which proved that caveolae had the ability to absorb the exogenous oils. Lastly, caveolin for oils absorption and lipase from *Bacillus pumilus* for oil hydrolysis were co-expressed in the naringenin-producing *Saccharomyces cerevisiae* strain, resulting in naringenin production increasing from 222 mg/g DCW (dry cell weight) (231 mg/L) to 269 mg/g DCW (241 mg/L). These results suggested that the caveolin-mediated transporter independent oil transport system would provide a promising strategy for the transport of hydrophobic substrates.

## 1 Introduction

High value-added nature products with a variety of chemical and biological functions can be produced by engineered microorganisms from plant oils and fatty acids [[1], [2], [3]]. The addition of fatty acid precursors was one of the simplest strategies to enhance fatty acid β-oxidation and promote the biosynthesis of acetyl-CoA derivatives. Zhang et al. demonstrated that the addition of fatty acids and the regulation of β-oxidation could improve the level of acetyl-CoA and promote the biosynthesis of naringenin [4]. The addition of fatty acids had many limitations such as toxicity to cells, destruction of cell membranes and reduction of intracellular pH [[5], [6], [7]] and the high price of the fatty acids was not suitable for industrial production. Some oils have a wide source and lower price compared with fatty acids, which meets the industrialization needs of low-cost production, they don't disturb the pH of the medium and destroy the cell membrane and will produce free fatty acids by hydrolysis by intracellular lipase after being transported into the cell. Therefore oils are expected to replace fatty acids as precursors to promote the efficient biosynthesis of acetyl-CoA derivatives.

Because of hydrophilic lipopolysaccharides on the cell membrane, the transport of larger hydrophobic substances into cells is severely limited [8,9]. As common hydrophobic substances, fatty acids mainly depend on fatty acid transporters into cells and FadL, a fatty acid transporter located in the outer membrane of *Escherichia coli*, was expressed to improve the fatty acid biotransformation [10]. In addition, the alkane transporter AlkL derived from *Pseudomonas putida* Gpo 1 could transport aliphatic alkanes C7 to C16 and fatty acid methyl esters into cells [11,12]. However, fatty acid transport requires transporters across the inner membrane through passive diffusion, which leads to a decrease in cell viability [13]. Only the transporter with the best expression level has high transport capacity [13] and this capacity limits the intracellular transport rate of fatty acids and other hydrophobic substances. As a potential substitute for fatty acids, if soybean oil is transported into cells by similar fatty acid transporters or hydrolyzed into fatty acids extracellularly then transported into cells, it would have the same disadvantages of fatty acid transport mentioned above. Therefore, mining an independent-transporters oil transport system, which can directly transport oils into cells, would avoid the problems caused by fatty acid addition and transport.

Caveolae, formed by caveolin encoding by *CAV1*, are membrane budding structures found in many vertebrate cells. One of the important functions of caveolae is to form membrane bending and endocytic vesicles, which play important roles in endocytosis, tumorigenesis and pathogen uptake [14]. The target substance is encapsulated in the caveolae to trigger the activation of the signaling cascade, which creates the fission and internalization of the caveolae and the release of the encapsulated substance [15]. Based on the function of endocytosis and release, caveolae had been used to engineered microorganisms. Shin et al. expressed caveolin in *E. coli*, making the strain more tolerant to toxic fatty acids and causing better growth ability, so that the biotransformation of the engineered strain from ricinoleic acid to (Z)-11-(heptanoyloxy) undec-9-enoic acid was 1.6 times higher than that of the control strain [13]. These results showed that caveolae-forming cells can rapidly transport fatty acids into cells, alleviate fatty acid-induced cytotoxicity, enhance the supply of acetyl-CoA and promote the biosynthesis of target products. This provided a potential strategy for the intracellular transport of other large hydrophobic substances.

In this study, the endocytosis function of caveolae was applied to *S. cerevisiae* to achieve the endocytosis of soybean oil, indicating that caveolae were the ideal hydrophobic substrate transport system. The caveolae structure in *S. cerevisiae* was first explored by the fusion expression of caveolin and green fluorescent protein (GFP). The ability of caveolae to transport exogenous substances was then verified with 5,6-carboxyfluorescein addition, indicating that the expression of *CAV1* could enhance the ability to endocytose exogenous substances. Soybean oil pre-stained with Nile Red was added to the medium. The result showed that the expression of *CAV1* could enhance the absorption of oils. Oils can be hydrolyzed to free fatty acids as the substrates of fatty acid β-oxidation by intracellular lipases, thus could promote acetyl-CoA biosynthesis by enhancement of fatty acid β-oxidation. Because the biosynthesis of one molecule of naringenin requires three molecules of acetyl-CoA and the low level of acetyl-CoA in cells limited the efficient biosynthesis of naringenin [16], the naringenin-producing *Saccharomyces cerevisiae* strain was used as the chassis cell to verify the effect of caveolin-enhanced oil absorption on fatty acid β-oxidation and biosynthesis of naringenin. After expressing *CAV1* and BP (lipase from *Bacillus pumilus*) in the strain, the production of naringenin was significantly increased. The results suggested that the co-expression of *CAV1* and lipase could enhance the absorption and hydrolysis of oil and promote the biosynthesis of naringenin in *S. cerevisiae*.

## 2 Materials and methods

### 2.1 Strains and plasmids

The engineered strain ZLHB04-4 (MATα, *ura3-52*, *trp1-289*, *leu2-3,112*, *his3Δ1*, *MAL2-8*^*c*^, *SUC2*, *gal80*:TRP1, *pdc5*:pGAL7-FjTAL, *aro 10*:pGAL1-ARO4^fbr^-pGAL10-ARO7^fbr^-G418 and rDNA:pGAL10-CHS-pALD5-CHI-pARO7-4CL-pTDH3-FjTAL) [17] generated from *S. cerevisiae* CEN. PK2-1D was used as the host for pathway engineering. The engineered strain C800 derived from *S. cerevisiae* CEN. PK2-1D (*gal80*:*KanMX*) was used as the host for functional identification of *CAV1* and *E*. *coli* JM109 was used for plasmid construction and storage. Plasmid pRS423 with *HIS3* selection was used to express target genes.

### 2.2 Strains and plasmids construction

The information of plasmids and primers used in this study are described in Table S1 and Table S2. Fragments of the *TGL1* and *TGL3* genes and promoter P_GAL7_ were amplified from the genomic DNA of *S. cerevisiae* S288c [18]. The DNA fragments of lipase from *Bacillus pumilus* (GenBank: JX163855) and human caveolin (GenBank: 403,908) were synthesized by Sangon Biotech Co., Ltd (Shanghai, China) after codon optimization. These sequences are shown in Table S3. The plasmids were constructed with a seamless cloning kit and all assembled plasmids were confirmed by Sanger sequencing before yeast transformation. Yeast transformations were conducted using the lithium acetate method [19,20] and the engineered recombinant strains are shown in Table 1.

**Table 1:** Strains used in this study.

Strain	Host strain	Description	Source
CEN.PK2-1D		MATα, *ura3-52*, *trp1-289*, *leu2*-*3,112*, *his3Δ1*, *MAL2*-8^C^, *SUC2*	[18]
ZLHB04-4	CEN.PK2-1D	Δ*gal80*::*TRP1*, Δpdc5::P_GAL7_-FjTAL, Δ*aro10*::P_GAL1_-*ARO4*^*fbr*^-P_GAL10_-*ARO7*^*fbr*^-G418, rDNA::P_GAL10_-CHS-P_ALD5_-CHI-P_ARO7_-4CL-P_TDH3_-FjTAL-*URA3*	[17]
L08	ZLHB04-4	pRS423-P_GAL7_-*CAV1*-T_TEF_	This study
L17	ZLHB04-4	pRS423-P_TEF_-BP-T_TEF_	This study
L18	ZLHB04-4	pRS423-P_TEF_-*TGL1*-T_TEF_	This study
L19	ZLHB04-4	pRS423-P_TEF_-*TGL3*-T_TEF_	This study
L20	ZLHB04-4	pRS423- P_GAL7_-*CAV1*-P_TEF_-BP	This study
L21	ZLHB04-4	pRS423-P_GAL7_-*CAV1*-P_TEF_-*TGL1*	This study
L22	ZLHB04-4	pRS423 P_GAL7_-*CAV1*-P_TEF_-*TGL3*	This study
C800	CEN.PK2-1D	*gal80*::*KanMX*	[43]
Z01	C800	pRS423-P_GAL7_-*CAV1*-T_TEF_	This study
Z04	C800	pRS423-P_GAL7_-GFP-T_TEF_	This study
Z10	C800	pRS423-P_GAL7_-*CAV1*-GFP-T_TEF_	This study
Z11	C800	pRS423-P_GAL7_-GFP-*CAV1*-T_TEF_	This study
Z12	C800	pRS423-P_GAL7_-*CAV1*-(GGGGS)_3_-GFP-T_TEF_	This study
Z13	C800	pRS423-P_GAL7_-GFP-(GGGGS)_3_-*CAV1*-T_TEF_	This study

### 2.3 Growth medium and shake-flask fermentation

*The E. coli* was cultured in Luria broth or agar plates and ampicillin at 100 μg/mL was added when required. The engineered *S. cerevisiae* strains were grown on synthetic dropout medium containing 1.74 g/L yeast nitrogen base (YNB) without amino acids (Sangon Biotech Co., Ltd), 20 g/L glucose, 5 g/L (NH_4_)_2_SO_4_ and 20 g/L agar was added when required, supplemented with appropriate amino acids including uracil 50 mg/L, histidine 50 mg/L, tryptophan 50 mg/L and leucine 50 mg/L depending on the auxotroph of the strains. The shake flask cultures were YPD medium prepared with 20 g/L glucose, 10 g/L yeast extract, 20 g/L peptone or YPDSO medium prepared with 20 g/L glucose, 10 g/L yeast extract, 20 g/L peptone, 1% [v/v] soybean oil and 2 g/L Tween 80.

For shake flask fermentation, the recombinant yeast was precultured in 5 mL of synthetic dropout medium on a shaker (Zhichu, Shanghai, China) (30 °C, 220 rpm). After 20 h growth, 300 μL of preculture was inoculated into 250 mL shake flask with 30 mL YPD or YPDSO medium for 72 h at 30 °C with shaking at 220 rpm. A microplate reader (synergyH1, Bio-Soar, Nanjing, China) was used to measure the cell density. All experiments were performed with biological replicates.

### 2.4 Analysis the structure and transportation of caveolae

For analysis of structure, the engineered strains Z01, Z10, Z11, Z12 and Z13 were precultured in 5 mL of YNB medium for 20 h. Precultured cells were inoculated into YNB medium and cultured for 72 h. The cells were harvested and washed four times with cold phosphate buffered saline (PBS) buffer and were resuspended in PBS buffer, then the cells were observed using fluorescence microscopy (Nikon, Tokyo, Janpan).

To analyze fluorescence dye transportation, the dye transport method was performed according to a previous method [14,21] The control strain C800 and the engineered strain Z01 were precultured in 5 mL of YNB medium for 20 h. Precultured cells were inoculated into YNB medium and cultured for 16 h then the strains were cultured for an additional 6 h after the addition of 10 mM 5,6-carboxyfluorescein. Cells were harvested and washed four times with cold PBS buffer until the fluorescent intensity of the supernatant reached blank level, then resuspended in PBS buffer and observed using laser confocal microscopy (Leica stellaris 5, Germany).

For analysis of hydrophobic substance transportation, the control strain C800 and engineered strain Z01 were precultured in 5 mL of YNB medium for 20 h. Precultured cells were inoculated into YPDSO medium pre-stained with Nile Red for 24 h. The cells were harvested and washed four times with cold PBS buffer until the fluorescent intensity of the supernatant reached blank level, then resuspended in PBS buffer and observed using laser confocal microscopy.

### 2.5 Lipid extraction and thin layer chromatography analysis

The control strain C800 and engineered strain Z01 were cultured in YPD supply with soybean oil (YPDSO) medium and 5 mL of fermentation broth and harvested after 48 h by centrifugation at 12,000×*g* for 15 min. The supernatants and pellets were used for lipid extraction and thin layer chromatographic (TLC) analysis [[22], [23], [24]]. The pellets were washed three times with distilled water (ddH_2_O) and extracted with 1 mL of 1:1 (v/v) methanol and chloroform supplemented with an internal standard of triheptadecanoylglycerol (Nu-chek-prep; Elysian, MN, USA). The mixture was then supplemented with 500 μL of ddH_2_O and centrifuged at 12,000×*g* for 15 min after vortexing for 15 min. The lower chloroform layer containing the lipids was transferred into a new 1.5 mL Eppendorf tube and extracted with 500 μL of 1:1 (v/v) methanol and chloroform by vortex for 15 min. The mixture had 250 μL of ddH_2_O added and was centrifuged at 12,000×*g* for 15 min after vortexing for 15 min. The chloroform extract was then subjected to TLC analysis. The supernatants were pretreated with 500 μL of acetic acid, 500 μL of 12% (w/v) sodium chloride solution and 2 mL of ethyl acetate, supplemented with an internal standard of triheptadecanoyl glycerol and vortexed for 15 min. The mixture was vortexed at 12,000×*g* for 15 min, then resuspended in chloroform for TLC analysis after the upper organic phase was collected and dried with nitrogen.

### 2.6 Metabolite quantification

Fermentation broth (500 μL) was mixed with methanol (500 μL) and vortexed vigorously for 5 min. Analysis and quantification were performed after centrifugation at 12,000×*g* for 6 min and 0.22 μm filtration. The supernatants were quantified through an high-performance liquid chromatography (HPLC) system (Shimadzu, Kyoto, Japan) equipped with a 250 × 4.6 mm, 5 μm C18 column (Thermo Fisher Scientific, Massachusetts, United States) [25]. The detection wavelength of (2*S*)-naringenin was 350 nm with an SPD-M20A UV detector (Shimadzu) at 40 °C. The samples were eluted with solvent A (water with 0.1% trifluoroacetic acid) and solvent B (acetonitrile with 0.1% trifluoroacetic acid) at a flow rate of 1 mL/min. The elution gradient was 10%–40% solvent B for 0–10 min, 40%–90% solvent B for 10–30 min, 90% solvent B for 30–35 min and 90%–10% solvent B for 30–35 min. To measure the soybean oil content, the supernatant was collected after centrifugation of culture broth, then drying in a conventional condition and measuring the weight of the soybean oil residue.

## 3 Results

### 3.1 The structure analysis of caveolin-mediated microvesicles

**Fig. 1:**
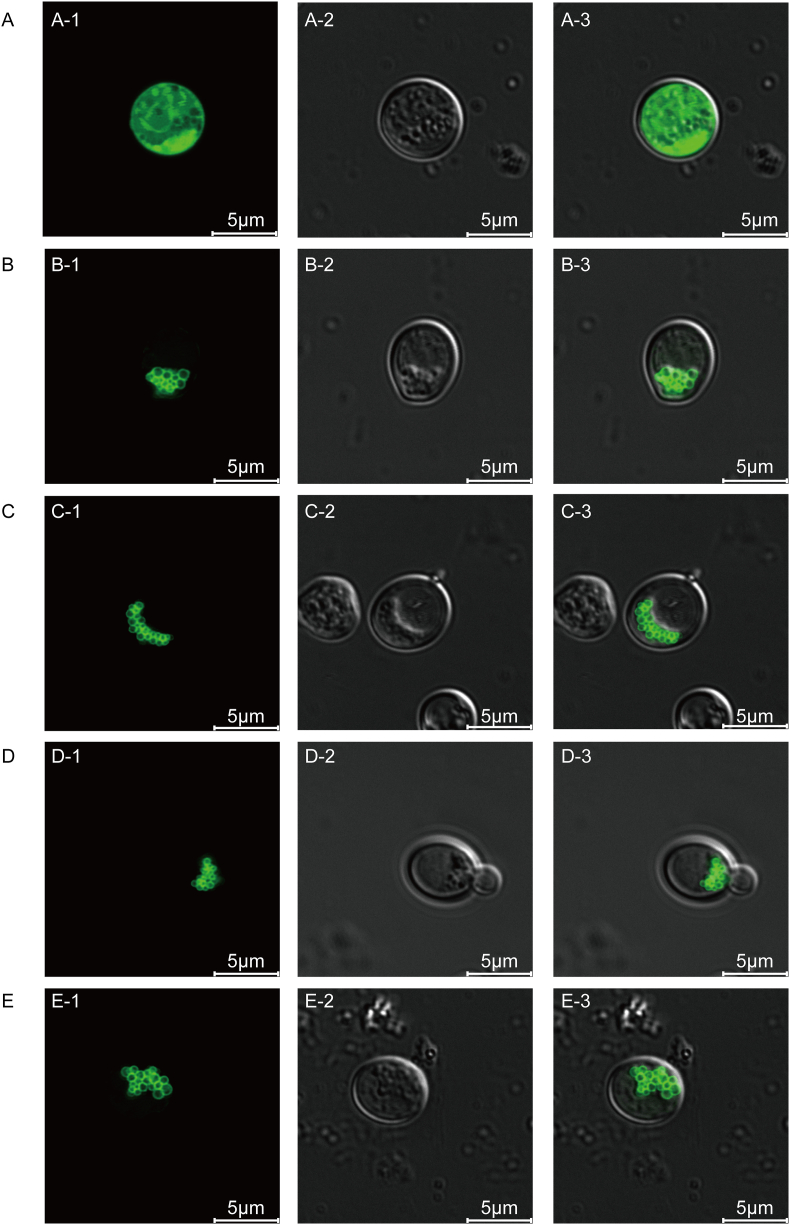
Confocal microscopy observation of caveolae formed in *S. cerevisiae.* Figure A, B, C, D and E: Representative images in control strain Z04, engineered strains Z10, Z11, Z12 and Z13, respectively. A-1, B-1, C-1, D-1 and E−1 are under fluorescence microscopy; A-2, B-2, C-2, D-2 and E−2 are under bright field microscopy; A-3, B-3, C-3, D-3 and E−3 are merged images of fluorescence microscopy and bright field microscopy.

The previous study showed that caveolin encoded by the *CAV1* gene could form caveolae in *E. coli* [13]. To analyze the structure of caveolin-mediated microvesicles and characterize the microvesicles structure in *S. cerevisiae*, the GFP was fused to the N-terminal or C-terminal of *CAV1*. As shown in Fig. 1, when the fluorescent protein was expressed alone, the fluorescence distribution filled the entire cell. While the fluorescent protein was fused with *CAV1*, the fluorescence formed small circular structures, suggesting that caveolin could mediate the formation of caveolae structure in *S. cerevisiae*. To further characterize the three-dimensional structure, the caveolae were observed by the Z-stack of the laser confocal microscope as small circles stacked together. There was no obvious effect on the structure of the caveolae when the green fluorescent protein was fused to the N-terminal or C-terminal of *CAV1* with or without a flexible linker.

### 3.2 The endocytosis of caveolae

**Fig. 2:**
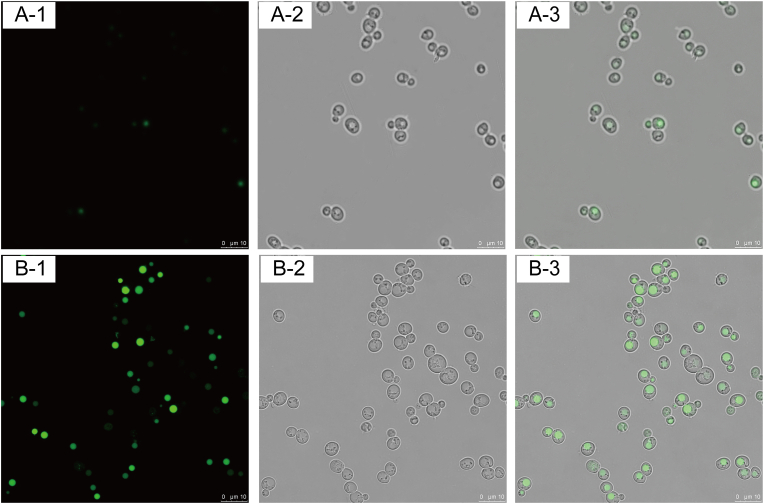
Endocytosis of 5,6-carboxyfluorescein in *S. cerevisiae.* A: The endocytosis of 5,6-carboxyfluorescein in control strain C800, A-1, A-2 and A-3 under fluorescence field microscopy, bright field microscopy and a merged image of the two, respectively. B: The endocytosis of 5,6-carboxyfluorescein in engineered caveolin-mediated strain Z01, B-1, B-2 and B-3 under fluorescence microscopy, bright field microscopy and a merged image of the two, respectively.

Caveolin mediated the formation of caveolae by invading the phospholipid bilayer membrane, which may be a transport carrier for endocytosis of substances in *S. cerevisiae*. To verify the uptake ability of caveolae, 5,6-carboxyfluorescein was used as the indicator and the intracellular fluorescence was observed using fluorescence microscopy (Fig. 2). The results showed that fluorescent intensity in the engineered strains was stronger than that of the control strain, indicating that the cells had a stronger ability to uptake 5,6-carboxyfluorescein with expression of *CAV1*. The weak fluorescence of the control strain may be due to the penetration of fluorescein into the cells and these results suggested that caveolae could strengthen the ability of taking up substances from the surrounding media.

### 3.3 Effect of engineered caveolae on the transportation of hydrophobic substances

**Fig. 3:**
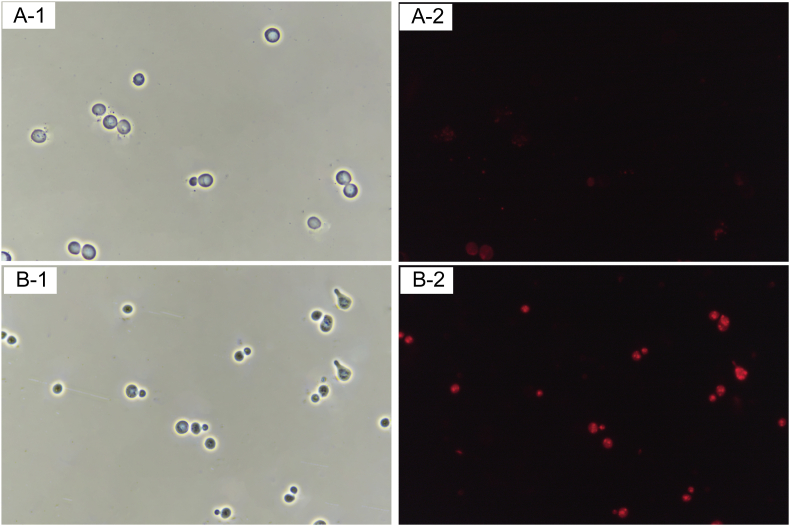
Endocytosis of hydrophobic substances in *S. cerevisiae*. A: The endocytosis of soybean oil stained with Nile Red in control strain C800, A-1 and A-2 under bright field and fluorescence microscopy. B: The endocytosis of soybean oil stained with Nile Red in engineered caveolin-mediated strain Z01, B-1 and B-2 under bright field and fluorescence field microscopy.

**Fig. 4:**
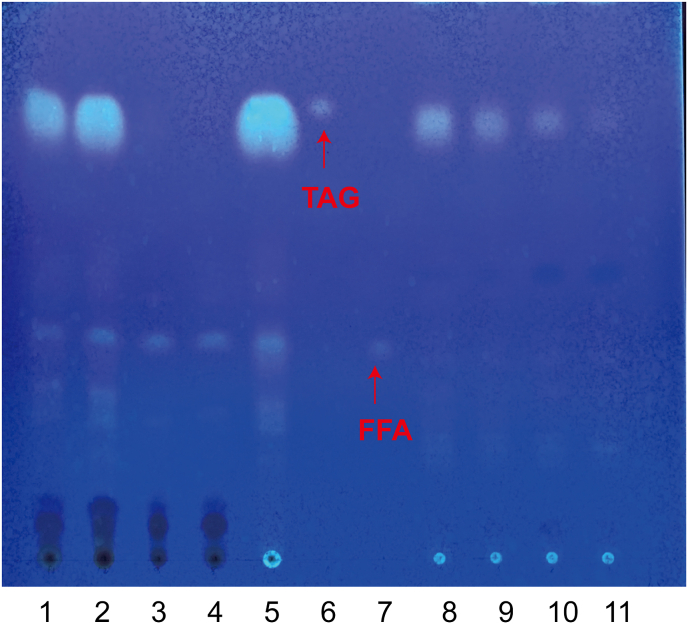
TLC analysis of intracellular and extracellular TAGs and FFAs. Lane 1: TLC analysis of extracellular lipids of engineered strain Z01 in YPDSO medium. Lane 2: TLC analysis of extracellular lipids of control strain C800 in YPDSO medium. Lane 3: TLC analysis of extracellular lipids of engineered strain Z01 in YPD medium. Lane 4: TLC analysis of extracellular lipids of control strain C800 in YPD medium. Lane 5: blank control of YPDSO medium; Lane 6: TAGs standard; Lane 7: free fatty acids (FFAs) standard. Lane 8: TLC analysis of intracellular lipids of engineered strain Z01 in YPDSO medium. Lane 9: TLC analysis of intracellular lipids of control strain C800 in YPDSO medium. Lane 10: TLC analysis of intracellular lipids of engineered strain Z01 in YPD medium. Lane 11: TLC analysis of intracellular lipids of control strain C800 in YPD medium.

To further verify the ability of caveolae to import extracellular oils, soybean oil was stained with Nile Red and then added to the culture medium. As shown in Fig. 3, the engineered cells showed red fluorescence due to oil absorption, while the control cells did not, indicating that caveolae could assist the absorption of exogenous oil droplets. Soybean oil is rich in triacylglycerol (TAG), which can be hydrolyzed by lipases to produce free fatty acids (FFAs) [26,27]. The intracellular and extracellular TAG and FFAs are detected by TLC to characterize the transport and absorption capacity of caveolin to soybean oil. As shown in Fig. 4, the extracellular TAG content of the *CAV1*-expressing cells in YPDSO medium was lower than that of the control cells and the intracellular TAG content of the *CAV1*-expressing cells was higher than that of the control cells, which further showed that caveolae could promote the absorption of oils. In YPD medium, the intracellular TAG content of the *CAV1*-expressing cells was equivalent to that of the control cells. There was no difference in the intracellular and extracellular FFAs content between the *CAV1*-expressing cells and the control cells in YPD or YPDSO media.

### 3.4 Effect of engineered caveolae and lipases on the production of (2*S*)-naringenin

**Fig. 5:**
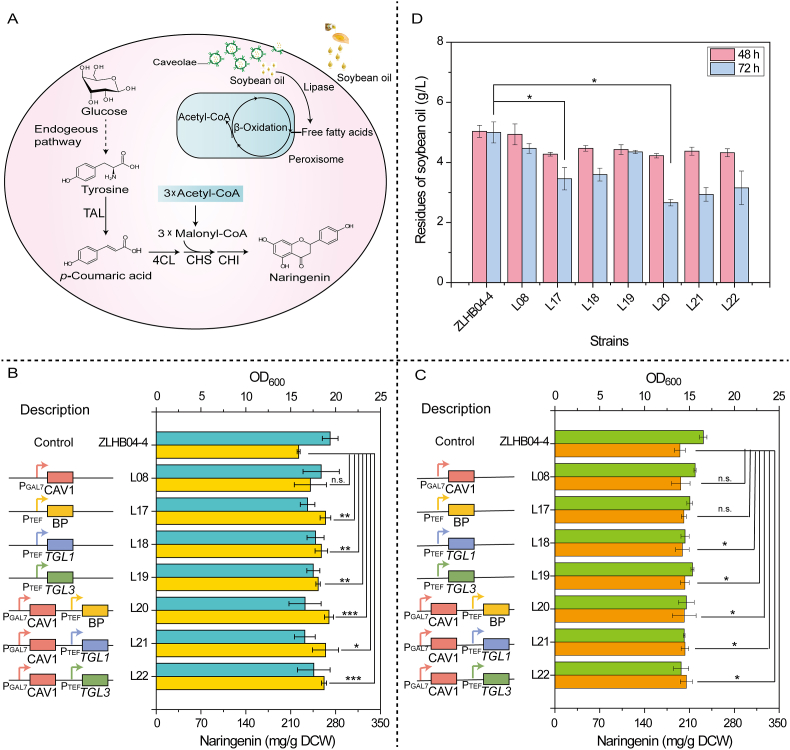
Effects of *CAV1* and lipases on naringenin production. A: The endocytosis process of soybean oil by caveolae and (2*S*)-naringenin biosynthetic pathway in *S. cerevisiae*. The dotted line represents multistep reactions. TAL, tyrosine ammonia lyase; 4CL, 4-hydroxycinnamoyl-CoA ligase; CHS, chalcone synthase; CHI, chalcone isomerase. B: The naringenin production of engineered strains in YPDSO medium. C: The naringenin production of engineered strains in YPD medium. BP: lipase from *Bacillus pumilus*, *TGL1*: the gene of sterol ester hydrolase from *S. cerevisiae*, *TGL3*: the gene of lipase from *S. cerevisiae*. Cyan columns represent OD_600_; yellow columns represent the production of naringenin. D: The residues of soybean oil in engineered strains. All data are means ± SD (n ≥ 3). *P < 0.05; **P < 0.01; ***P < 0.001; n. s. *P* > 0.05. (Student's *t*-test: two-tailed, two-sample equal variance).

Caveolae mediated by caveolin possess a transport function that does not depend on transporters and was expected to enhance the endocytosis and hydrolysis of exogenous oil through the overexpression of *CAV1* and/or lipase genes, to promote fatty acid β-oxidation (Fig. 5A). As shown in Fig. 5B, in YPDSO medium, the naringenin production of the engineered strain L08 with the overexpression of *CAV1* at 240 mg/g DCW was higher than that of the control strain at 222 mg/g DCW, indicating that the overexpression of *CAV1* could promote the transport of oils into cells and improve the production of naringenin, an acetyl-CoA derivative.

To further promote the hydrolysis of endocytosed oils and enhance the process of β-oxidation, lipase from *Bacillus pumilus* (BP), lipase from *S. cerevisiae* (encoded by *TGL3*) and sterol ester hydrolase from *S. cerevisiae* (encoded by *TGL1*) were overexpressed. The results showed that the production of naringenin in engineered strains were higher than that of the control strain regardless of whether lipases were overexpressed alone (engineered strains L17, L18 and L19) or co-overexpressed with *CAV1* (engineered strain L20, L21 and L22), indicating that lipases could promote the biosynthesis of naringenin in YPDSO medium as seen in Fig. 5B. The naringenin production reached 262 mg/g DCW in engineered strain L22 expressing *TGL3* and *CAV1*, which was significantly higher than that of engineered strain L19 with the overexpression of *TGL3* at 252 mg/g DCW. Compared with the production of naringenin of engineered strain L17 at 264 mg/g DCW with the overexpression of BP lipase, the production of naringenin of engineered strain L20 with the co-overexpression of BP and *CAV1* increased to 269 mg/g DCW. Similarly, the production of naringenin of engineered strain L21 with co-overexpression of *TGL1* and *CAV1* was 264 mg/g DCW, but the naringenin production of engineered strain L18 with the overexpression of *TGL1* was 257 mg/g DCW. Different from the effect in YPDSO medium, there was no significant difference in naringenin production between the engineered strains and the control strain in YPD medium (Fig. 5C). Moreover, the extracellular soybean oil content of the engineered strains was lower than that of the control strain, which proved that the absorption and utilization capacity of soybean oil by the engineered strains was better than that of the control strain (Fig. 5D). It was concluded that the biosynthesis of acetyl-CoA derivatives was promoted by enhancing the expression of caveolin and lipases supplied with soybean oil supply. However, biomass of engineered strains were lower than that of control strain, which may be due to the endocytosis of soybean oil and/or the metabolic burden caused by heterologous genes overexpression and product biosynthesis.

## 4 Discussion

Fatty acid transport requires the participation of fatty acid transporters, but the transport of fatty acids across the cell membrane into the cytosol impairs the integrity of the membrane and limits the availability of substrates [28,29], so an ideal transport system independent of fatty acid transporters is required to improve the transport of hydrophobic substrates. In this study, the structure of caveolae in *S. cerevisiae* was explored by expressing the *CAV1* gene derived from humans. It was found that caveolae in *S. cerevisiae* formed a hollow structure with the scattered accumulation of caves. Fluorescein and soybean oil in the media were transported into cells, which proved the endocytosis function of caveolae. The caveolae were then applied to the naringenin-producing strains and promoted soybean oil transportation into cells to enhance fatty acid β-oxidation and increased naringenin production. The expression of lipases also promoted the hydrolysis of intracellular soybean oil and improved the biosynthesis of naringenin, providing an additional strategy for enhancing the supply of acetyl-CoA and promoting the biosynthesis of acetyl-CoA derivatives.

The caveolae are flask-like structures with invaginated plasma membranes in humans [15,30]. After the *CAV1* gene derived from humans was expressed in *E. coli*, circular caveolae were detected near the cytoplasmic membrane [13]. These studies showed that caveolae had endocytic function both in humans and *E. coli,* so they were expected to have the same function in *S. cerevisiae*. However, the structure and the endocytosis function of caveolae were unclear in *S. cerevisiae*, but by fusing *CAV1* with green fluorescent protein, the structure of caveolae was constructed and analyzed, showing that caveolae formed a hollow structure. To identify the endocytosis of caveolae in *S. cerevisiae,* 5,6-carboxyfluorescein was supplemented in the media and the fluorescence intensity of *CAV1*-expressing cells was significantly higher than that of the control, suggesting that expression of *CAV1* gene could promote the transport of exogenous substrates in *S. cerevisiae.* This agreed with previous studies, which showed that expression of *CAV1* in *E. coli* formed caveolae to facilitate 5,6-carboxyfluorescein to transport into cells [13,14]. Soybean oil that was pre-stained with Nile Red, was used to show that the caveolae formed in *S. cerevisiae* had the ability to transport hydrophobic substrates and these results confirmed that the expression of *CAV1* had endocytic function in microorganisms such as *E. coli* and *S. cerevisiae*.

In *S. cerevisiae*, tyrosine undergoes deamination, ligation, condensation and isomerization to form naringenin [16,31]. The condensation reaction by chalcone synthase (CHS) is the key rate-limiting step [32,33]. Previous studies showed that strengthening the activity of CHS can promote the biosynthesis of naringenin [[34], [35], [36]]. However, the condensation reaction also required three molecules of acetyl-CoA as precursors, which made the supply of acetyl-CoA as another limiting factor [37]. In *S. cerevisiae*, fatty acid β-oxidation in peroxisomes is an important source of acetyl-CoA [4,38,39]. This study aimed to strengthen fatty acid β-oxidation by adding oils instead of adding fatty acids, to promote the supply of acetyl-CoA. As expected, after the caveolae transported soybean oil into cells, the yield of naringenin significantly increased from 222 mg/g DCW in control strain ZLHB04-4 to 269 mg/g DCW in the engineered strain L20. The co-expression of caveolin and BP lipase resulted in the increase of naringenin production, which was an important example of caveolae completing oil endocytosis and achieving the biosynthesis of high-value acetyl-CoA derivatives. Although the production of naringenin only slightly increased from 231 mg/L in control strain ZLHB04-4 to 241 mg/L in the engineered strain L20 (data not shown), which may be due to the insufficient downstream pulling force of fatty acid β-oxidation. Therefore, the next work is to overexpress key genes of fatty acid β-oxidation to further strengthen fatty acid β-oxidation. In addition, *CAV1* was expressed by different promoters to analyze the effect of different expression levels of caveolin on naringenin production. Opposite to the effect of strong promoter (pGAL1), the naringenin production was no significant difference when *CAV1* was expressed with medium and weak promoters (data not shown). Therefore, the strong promoter was more conducive to the expression of *CAV1*, which may be due to the formation of more microvesicles to transport and absorb soybean oil.

Compared with soybean oil, the waste oil produced in industry and life (such as waste cooking oil or leaked crude oil [40]) is not friendly to the environment and food safety [41,42]. Therefore, exploring the utilization of waste oil and crude oil to produce high value-added biochemical products is crucial. In this study, caveolae-forming *S. cerevisiae* had the ability to absorb soybean oil. If caveolae-forming *S. cerevisiae* was applied for endocytosis of these waste and crude oil to promote high value-added acetyl-CoA derivatives production, it would not only address the problems of waste oil reprocessing and crude oil spilling, but also promote the production of high-value products. The endocytosis system is expected to become a green tool to solve environmental problems and achieve resource reuse to produce high value-added products.

## CRediT authorship contribution statement

**Qian Zhang:** Conceptualization, the study, Methodology, performed the experiments, Formal analysis, Writing – original draft, designed and coordinated the project. **Ning Li:** Conceptualization, the study, Writing – original draft, designed and coordinated the project. **Yunbin Lyv:** Conceptualization, the study, performed the experiments, Formal analysis, designed and coordinated the project. **Shiqin Yu:** conceived the study, designed and coordinated the project. **Jingwen Zhou:** Conceptualization, the study, Methodology, Writing – original draft, designed and coordinated the project, All authors discussed the results and commented on the manuscript.

## Declaration of competing interest

The authors declare there is no conflict of interest.
