# Supplementary material for: Engineering caveolin-mediated endocytosis in Saccharomyces cerevisiae
Source: Synth Syst Biotechnol. 2022 Jul 2;7(4):1056–63. doi: 10.1016/j.synbio.2022.06.008 (PMC9263866; doi:10.1016/j.synbio.2022.06.008)
Supplement: Multimedia component 1 [file mmc1.docx]

**Supplementary materials**

**Table S1. Plasmids used in this study**

| **Plasmid** | **Description** |
| --- | --- |
| P01 | pRS423-P_GAL7_-*CAV1*-T_TEF_ |
| P02 | pRS423-P_TEF_-BP-T_TEF_ |
| P03 | pRS423-P_TEF_-*TGL1*-T_TEF_ |
| P04 | pRS423-P_TEF_-*TGL3*-T_TEF_ |
| P05 | pRS423- P_GAL7_-*CAV1*-P_TEF_-BP |
| P06 | pRS423-P_GAL7_-*CAV1*-P_TEF_-*TGL1* |
| P07 | pRS423 P_GAL7_-*CAV1*-P_TEF_-*TGL3* |
| P08 | pRS423-P_GAL7_-*CAV1*-GFP-T_TEF_ |
| P09 | pRS423-P_GAL7_-GFP-*CAV1*-T_TEF_ |
| P10 | pRS423-P_GAL7_-*CAV1*-(GGGGS)_3_-GFP-T_TEF_ |
| P11 | pRS423-P_GAL7_-GFP-(GGGGS)_3_-*CAV1*-T_TEF_ |

**Table S2. Primers used in this study**

| **Name** | **Sequence (5ʹ-3ʹ) ^*^** |
| --- | --- |
| P7-423-F | CACCAGACATTTTTGAGGGAATATTCAACTGTTTTTTTTTATCATGTTGATG |
| P7-423-R | ACAAAAGCTGTTTGCCAGCTTACTATCCTTCTTGAAAATATGC |
| CAV1-423-F | TGACTCGAGTTAAATTTCCTTTTGCAAATTAATTCTAACGTTAGAGAAAATTTTACCAA |
| CAV1-423-R | TCCCTCAAAAATGTCTGGTGGTAAATACGTTGATTCTGAAG |
| 423-P7CAV1-F | AGCTGGCAAACAGCTTTTGTTCCCTTTAGTGAGGGTTAATTG |
| 423-P7CAV1-R | GGAAATTTAACTCGAGTCATGTAATTAGTTATGTCACGC |
| BP-423-F | ATGACTCGAGTTAGTTTGTATTTTGGCCGCCACC |
| BP-423-R | TAGAACTAGTATGAAAGTGATGTTTGTTAAGAAAAATGAGTTTGC |
| 423-BP-F | TCACTTTCATACTAGTTCTAGAAAACTTAGATTAGATTGCTATGCTTTCTTTC |
| 423-BP-R | TACAAACTAACTCGAGTCATGTAATTAGTTATGTCACGCTTAC |
| TGL1-423-F | ATGACTCGAGTCATTCTTTATTTAGAGCATCCAGCGCAG |
| TGL1-423-R | TAGAACTAGTATGTACTTCCCCTTTTTAGGCAGATTATCG |
| 423-TGL1-F | GGAAGTACATACTAGTTCTAGAAAACTTAGATTAGATTGCTATGCTTTC |
| 423-TGL1-R | TAAAGAATGACTCGAGTCATGTAATTAGTTATGTCACGCTTAC |
| TGL3-423-F | ATGACTCGAGCTACCTACTCCGTCTTGCTCTTATTATGTCG |
| TGL3-423-R | TAGAACTAGTATGAAGGAAACGGCGCAGGAATA |
| 423-TGL3-F | TTTCCTTCATACTAGTTCTAGAAAACTTAGATTAGATTGCTATGCTTTC |
| 423-TGL3-R | GAGTAGGTAGCTCGAGTCATGTAATTAGTTATGTCACGCTT |
| Ter-423CAV1-F | GGGCGAATTGGAGAGTAGACTTTTTCTGTGAAATTTAATGAGTTTTTGTTC |
| Ter-423CAV1-R | GGAAATTTAATCACATATGAAAGTATATACCCGCTTTTGTACAC |
| 423CAV1-Ter-F | TCATATGTGATTAAATTTCCTTTTGCAAATTAATTCTAACGTTAGAGAAAATTTTACC |
| 423CAV1-Ter-R | GTCTACTCTCCAATTCGCCCTATAGTGAGTCGTATTACG |
| PBP-423CAV1ter-F | GGGCGAATTGGAGCTCATAGCTTCAAAATGTTTCTACTCCTTTTTTAC |
| PBP-423CAV1ter-R | GTCTACTCTCTTAGTTTGTATTTTGGCCGCCACCAT |
| 423CAV1ter- PBP-F | TACAAACTAAGAGAGTAGACTTTTTCTGTGAAATTTAATGAGTTTTTGTTC |
| 423CAV1ter- PBP-R | CTATGAGCTCCAATTCGCCCTATAGTGAGTCGTATTACG |
| PTGL1-423CAV1ter-F | GGGCGAATTGGAGCTCATAGCTTCAAAATGTTTCTACTCCTTTTTTAC |
| PTGL1-423CAV1ter-R | GTCTACTCTCTCATTCTTTATTTAGAGCATCCAGCGCAG |
| 423CAV1ter- PTGL1-F | ATAAAGAATGAGAGAGTAGACTTTTTCTGTGAAATTTAATGAGTTTTTGTTC |
| 423CAV1ter- PTGL1-R | CTATGAGCTCCAATTCGCCCTATAGTGAGTCGTATTACG |
| PTGL3-423CAV1ter-F | GGGCGAATTGGAGCTCATAGCTTCAAAATGTTTCTACTCCTTTTTTAC |
| PTGL3-423CAV1ter-R | GTCTACTCTCCTACCTACTCCGTCTTGCTCTTATTATGTCG |
| 423CAV1ter- PTGL3-F | GAGTAGGTAGGAGAGTAGACTTTTTCTGTGAAATTTAATGAGTTTTTGTTC |
| 423CAV1ter- PTGL3-R | CTATGAGCTCCAATTCGCCCTATAGTGAGTCGTATTACG |
| GFP-P08-F | ATTACATGACTCGAGTTATTTGTATAGTTCATCCATGCCATGTGTAATCC |
| GFP-P08-R | TTGCAAAAGGAAATTATGGGTAAGGGAGAAGAACTTTTCACTG |
| P08-GFP-F | TTCTCCCTTACCCATAATTTCCTTTTGCAAATTAATTCTAACGTTAGAGAAAATTTTAC |
| P08-GFP-R | GAACTATACAAATAACTCGAGTCATGTAATTAGTTATGTCACGC |
| GFP-P09-F | TTTACCACCAGACATTTTGTATAGTTCATCCATGCCATGTGTAATCC |
| GFP-P09-R | AATATTCCCTCAAAAATGGGTAAGGGAGAAGAACTTTTCACTG |
| P09-GFP-F | TTCTCCCTTACCCATTTTTGAGGGAATATTCAACTGTTTTTTTTTATCATGTTGATG |
| P09-GFP-R | GATGAACTATACAAAATGTCTGGTGGTAAATACGTTGATTCTGAAG |
| GFP-P10-F | ATTACATGACTCGAGTTATTTGTATAGTTCATCCATGCCATGTGTAATCC |
| GFP-P10-R | GGAGGTGGTGGTAGTGGAGGTGGAGGGAGTGGTGGTGGTGGCAGTATGGGTAAGGGAGAAGAACTTTTCACTG |
| P10-GFP-F | ACTGCCACCACCACCACTCCCTCCACCTCCACTACCACCACCTCCAATTTCCTTTTGCAAATTAATTCTAACGTTAGAGAAAATTTTAC |
| P10-GFP-R | AACTATACAAATAACTCGAGTCATGTAATTAGTTATGTCACGC |
| GFP-P11-F | ACTGCCACCACCACCACTCCCTCCACCTCCACTACCACCACCTCCTTTGTATAGTTCATCCATGCCATGTGTAATCC |
| GFP-P11-R | AATATTCCCTCAAAAATGGGTAAGGGAGAAGAACTTTTCACTG |
| P11-GFP-F | TTCTCCCTTACCCATTTTTGAGGGAATATTCAACTGTTTTTTTTTATCATGTTGATG |
| P11-GFP-R | GGAGGTGGTGGTAGTGGAGGTGGAGGGAGTGGTGGTGGTGGCAGTATGTCTGGTGGTAAATACGTTGATTCTGAAG |

*Underlined sequences are homologous arm sequences.

**Table S3. Synthesized lipase gene**

| **Source** | **GenBank Accession No.** | **Codon Optimized Sequence** |
| --- | --- | --- |
| *Bacillus pumilus* | JX163855 | ATGAAAGTGATGTTTGTTAAGAAAAATGAGTTTGCCATACTAATCGCGTTAGCCTTGGTTATCGGAAGTATGGCCTTCATCCAACCTAAGGAGGTTAAAGCCGCAGAGCACAATCCAGTGGTCATGGTCCACGGGATGGGAAGTGGTAGCTATAATTTCGCAAGTATAAAGAACTACTTAATAGGCCAGGGCTGGGATAGGAACCAACTGTATGCTATAGATTTCATTGATAAAACAGGCAACAATAGAAATAATGGCCCTCGTTTGAGCAGATTCGTCAAGGATGTGCTTGACAAGACAGGAGCCAAGAAAGTAGACATCGTCGCCCATTCAATGGGAGGTGCCAATACATTATATTATATCAAGAACTTGGACGGCGGGGACAAGATTGAGAATGTCGTGACAATCGGAGGGGCGAACGGGTTGTCATCCAGTAGGGCATTGCCAGGTACAGATCCCAATCAGAAGATTCTTTACACTTCTGTTTATTCCAGCGCGGATCTAATCGTGGTTAACTCACTATCACGTTTAATCGGAGCTAGAAACATACTTATACATGGGGTGGGACATATCGGCTTGCTTACAAGTTCCCAGGTAAAGGGTTACGTCAAAGAAGGCTTGAATGGTGGCGGCCAAAATACAAACTAA |
| CAV1 | 403908 | ATGTCTGGTGGTAAATACGTTGATTCTGAAGGTCATTTGTACACTGTTCCAATTAGAGAACAAGGTAACATCTACAAGCCAAACAACAAGGCTATGGCTGATGAATTGTCTGAAAAGCAAGTTTACGATGCTCATACTAAGGAAATTGATTTGGTTAACAGAGATCCAAAGCACTTGAACGATGATGTTGTTAAGATTGATTTCGAAGATGTTATTGCTGAACCAGAAGGTACTCATTCTTTCGATGGTATTTGGAAGGCTTCTTTCACTACTTTCACTGTTACTAAGTACTGGTTCTACAGATTGTTGTCTGCTTTATTCGGTATTCCAATGGCTTTGATTTGGGGTATCTATTTCGCTATTTTGTCCTTTTTGCATATTTGGGCTGTTGTTCCATGTATTAAGTCTTTCTTGATTGAAATTCAATGTATTTCTAGAGTTTACTCTATCTACGTTCATACTGTTTGTGATCCATTGTTTGAAGCTGTTGGTAAAATTTTCTCTAACGTTAGAATTAATTTGCAAAAGGAAATTTAA |
